# The Role of Sensory Impairments on Recovery and Rehabilitation After Stroke

**DOI:** 10.1007/s11910-025-01407-9

**Published:** 2025-03-06

**Authors:** Joanna E. Hoh, Jennifer A. Semrau

**Affiliations:** 1 Department of Kinesiology and Applied Physiology, University of Delaware, Newark, DE USA; 2 Interdisciplinary Graduate Program in Biomechanics and Movement Science, University of Delaware, Newark, DE USA; 3 Department of Biomedical Engineering, University of Delaware, Newark, DE USA

**Keywords:** Somatosensation, Stroke, Recovery, Neurorehabilitation, Proprioception, Tactile

## Abstract

**Purpose of Review:**

The current review aims to address critical gaps in the field of stroke rehabilitation related to sensory impairment. Here, we examine the role and importance of sensation throughout recovery of neural injury, potential clinical and experimental approaches for improving sensory function, and mechanism-based theories that may facilitate the design of sensory-based approaches for the rehabilitation of somatosensation.

**Recent Findings:**

Recently, the field of neurorehabilitation has shifted to using more quantitative and sensitive measures to more accurately capture sensory function in stroke and other neurological populations. These approaches have laid the groundwork for understanding how sensory impairments impact overall function after stroke. However, there is less consensus on which interventions are effective for remediating sensory function, with approaches that vary from clinical re-training, robotics, and sensory stimulation interventions.

**Summary:**

Current evidence has found that sensory and motor systems are interdependent, but commonly have independent recovery trajectories after stroke. Therefore, it is imperative to assess somatosensory function in order to guide rehabilitation outcomes and trajectory. Overall, considerable work in the field still remains, as there is limited evidence for purported mechanisms of sensory recovery, promising early-stage work that focuses on sensory training, and a considerable evidence-practice gap related to clinical sensory rehabilitation.

## Introduction

In everyday life, our daily activities involve integrating somatosensory information across a variety of sources. A common example of this is eating, which includes grasping a fork, coordinating movements between the hands to cut a piece of food with a fork and knife, and then bringing the food to our mouths. Using touch, we are able to modulate applied pressure to properly grasp and manipulate the fork or to pick up a piece of food off the plate with our fingers. Using proprioception, or our sense of body position and movement, we are able to plan how we will move our fork to obtain the piece of food, use feedback to modify movements, and refine our executed return movement to eat the piece of food. Notably, we are typically able to perform this sequence of movements seamlessly using various sub-modalities of somatosensation, which include touch, proprioception, temperature, and pain. However, when neural injury or damage occurs, as in stroke, the ability to use somatosensory information to guide movement can be significantly diminished and can significantly impact everyday function.

Overall, the somatosensory system is critical for skilled movements that we use to complete daily activities. A significant body of research in neurologically intact controls and clinical populations has highlighted the critical role of somatosensation (or sensory) function for movement planning, online control, and execution. Sensory function is essential for providing feedback on action-based movement execution – including grasping and interacting with everyday objects [1], refining how we move in smooth, controlled ways [2], and responding quickly to stimuli with protective responses for safety [3]. Prior work focusing on the impact of sensory impairments on movement has primarily focused on individuals with sensory deafferentation. These individuals have little or no afferent limb information that is communicated from the periphery to the brain [2, 4–6]. In this rare, small population, upper limb movements are typically “normal” when the individual has vision of the arm and hand, but become less accurate and more variable when vision is occluded [7]. Importantly, this clinical work has established that intact afferent signaling is necessary for movement planning and execution [8]. While these studies have been critically important for understanding the contributions of sensory control for proper movement, they focus primarily on the disruption of peripheral signaling of somatosensory information rather than the impacts of central or brain injury which are far more common in neurologic conditions.

In centrally occurring neural injury or disease, including stroke and Parkinson’s disease, aspects of sensory function are commonly affected, resulting in diminished or impaired somatosensation. While somatosensation includes the sub-modalities of touch, proprioception, pain, and temperature – not all individuals with neural injury experience deficits in all four domains. Across each of the modalities of somatosensation, reported incidence of impairment after stroke has been highly variable with reports of tactile sensation impairments ranging from 30 to 62% [9–14], pain-related impairments ranging from 1 to 12% [15], and impairments in proprioception ranging from 25 to 80% [9, 10, 12, 16–19]. Clinically, sensory deficits can present as poor quality of movement such as difficulty with graded force control [20, 21], coordination [22], balance [23–26], and activities of daily living (ADL) [10, 19]. Based on the widespread impact of the clinical presentation of sensory deficits, it is unsurprising that sensory deficits can have a significant impact on independence and quality of life [19, 22]. To date, it has been recognized that assessments and diagnosis of sensory impairments are important for understanding patient status upon initiating rehabilitation [27] and some studies have shown that sensory status is an important part of recovery and is potentially a predictor of overall/motor outcomes after stroke [28].

Currently, a significant knowledge gap exists in understanding how sensory impairments impact motor and functional recovery after stroke, as well as limited knowledge related to how neurorehabilitation can effectively improve somatosensation. The current review seeks to address these critical gaps by examining the importance of somatosensation for recovery of neural injury, potential therapies for improving sensory function, and visiting mechanism-based theories that may facilitate the design of sensory-based approaches for the rehabilitation of somatosensation. While the deleterious effects of several neurologic diseases and injuries have been reported for somatosensation [26, 29, 30], this review will primarily focus on stroke since the majority of current work focusing on sensory recovery and rehabilitation has been studied in stroke survivors.

### Critical Interdependence of Motor and Somatosensory Systems for Recovery

In neurorehabilitation, a primary goal of recovery is the return or improvement of motor function of the upper and/or lower limbs. Motor impairments are exceedingly common after stroke [31] and are typically the focus of the rehabilitation process. Despite the attention motor recovery receives in neurorehabilitation practice and research, clinical practice guidelines for stroke include little information regarding somatosensory assessment, and even less on intervention and recovery [32–35]. Additionally, research in stroke rehabilitation has only begun to seriously focus on post-stroke somatosensory impairments in the past 10–15 years. This is surprising given the wide range of somatosensory deficits that are reported after stroke and the interconnectedness of the motor and somatosensory systems [36]. To date, it is estimated that ~ 50% of individuals have a deficit in at least one sub-modality of somatosensation after stroke [3, 14].

Previous work in the field of motor control has demonstrated the interconnectedness of the somatosensory and motor systems, particularly for touch and proprioception [2, 4, 37–39]. When the somatosensory and motor systems are intact and able to appropriately integrate information to guide action, we are able to complete accurate and purposeful movements that support everyday function. However, individuals with stroke often have deficits in the somatosensory system, motor system, or combined deficits in both systems. The problem of understanding sensory function, particularly proprioception, has been especially challenging to investigate as it is difficult to disentangle sensory and motor deficits when movement execution relies heavily on both systems. Therefore, assessments or interventions targeting either the somatosensory or motor systems independently need to carefully consider design or approach to isolate the system of interest. Isolation of specific deficits is a fundamental difficulty for both sensorimotor assessment and training paradigms, particularly those with outcomes focused on improving motor function, as the somatosensory system continually contributes feedback about estimates of limb state. This begs the question about whether any motor interventions are truly devoid of sensory training, which suggests that it is critical to not only assess but understand the contributions of sensory function during motor intervention.

### Current Approaches for Assessment of Somatosensory Function

To date, sensation is rarely assessed in clinical environments, and when tested, tends to be a small portion of the typical neurological exam [40, 41]. A current limitation within clinical care is the general absence of assessments that aim to accurately and objectively quantify somatosensory impairments. Clinical assessments like the Rivermead Assessment of Somatosensory Performance (RASP) [42] or the revised Nottingham Sensory Assessment (NSA) [43, 44]. are not typically used for day-to-day clinical evaluations [40], but are used to quantify somatosensory outcomes in research studies. This utilization is similar to how the Fugl-Meyer is used to quantify outcomes of motor recovery after stroke in clinical trials [45–47]. Recent work has found that clinicians are more familiar with non-standardized somatosensory assessments, especially in the tactile domain of somatosensation, which fails to address critical aspects of sensorimotor impairment that affects daily function, like proprioception [40, 48, 49]. In a recent study from our group, we found that clinicians reported using a non-standardized assessment of light touch 55% of the time during stroke evaluations, compared to 25% and 0% on the proprioceptive up/down and mirror position tests, respectively [40]. Additionally, clinicians reported prioritizing time during evaluation for motor over somatosensory assessments for in-clinic assessments after stroke [40]. Given the critical links between sensory and motor function for movement production, it is surprising that standardized tests of somatosensation have significantly lagged behind those for motor function after stroke. While clinicians seem to prioritize tactile assessments during evaluations, it is important to note that findings in the tactile domain may not inform assessments in other somatosensory domains, such as proprioception. Therefore, it is critical to implement multi-modality testing of somatosensory deficits to appropriately inform treatment [13, 50]. Additionally, while assessments for proprioception and tactile function have been developed and implemented for research studies, there still remains a considerable evidence-practice gap between current research and clinical implementation [41, 48, 51].

In the past decade, advances using technology-based and robotic assessments have proven useful for precise identification of somatosensory impairments, namely proprioception, in a variety of neurologic populations for both the upper and lower limbs [16, 17, 52–55]. The use of technology aids in reducing confounds, such as tactile contact from assessors, poor intra- or inter-rater reliability, and is beneficial for standardizing protocols and measurement approaches. The majority of recent technology-based approaches have focused on limb proprioception, with fewer studies using advanced methods to evaluate tactile sensation. A recent study examining tactile sensation used robotics to simulate real-world sensations like brushing against the skin or mechanical stretch of the skin, a promising technique to effectively assess the perception of touch [56]. Additionally, perceptual thresholds of tactile sensation have been assessed in stroke survivors via electrical stimulation, a technique which has demonstrated good feasibility and intra-rater reliability [57].

In comparison, technology-based approaches for measuring proprioception after stroke have been more numerous in recent years. Much of this work has been inspired by previous work by Dr. Leeanne Carey and colleagues, as it was some of the first to target and develop objective assessments for somatosensory impairments in stroke survivors [58–61]. Within the past decade, our group and others have used technology-based assessments to measure somatosensory deficits as they capture continuous, rather than ordinal, metrics [62]. This work has been critical for beginning to understand sensory impairments after stroke, as it has developed new techniques for evaluating proprioception [16, 17, 63, 64], identified differences in impairment across sub-domains of proprioception [65], shown methodological differences when comparing unilateral versus bilateral testing [52], and has begun to better understand the underlying neural mechanisms responsible for impaired proprioception after stroke [28, 66–74].

### Novel Approaches to Sensory Training and Intervention

A current question that remains unanswered in the field is whether it is necessary to “retrain” sensory function. Some evidence points to positive effects for sensory recovery from either movement or sensorimotor-based interventions. For example, the Video Game Rehabilitation for Outpatient Stroke (VIGoROUS) trial was created as a gamified constraint-induced movement therapy (CIMT) intervention [75]. Secondary outcomes found that tactile sensation spontaneously recovered concomitantly with motor function, suggesting a link between the recovery of tactile sensory function and motor function [76]. However, other studies have found that recovery of sensory and motor function is mismatched for ~ 30% of individuals while receiving standard of care rehabilitation [77]. This contrast in findings serves as a reminder that while spontaneous sensory recovery may appear similar to that observed for motor recovery [78], the mechanisms and/or timeline of recovery between sensory and motor function may not be identical, but as recent work has shown, sensory recovery is likely required for full functional recovery [79].

Given that current research has established that there is reliance on proprioception and tactile sensation for motor execution and motor learning [80–82], it is reasonable to think that training impaired aspects of sensation would benefit motor outcomes during recovery. Despite this, in comparison to motor training and rehabilitation studies, there have been relatively few studies that directly target sensory retraining. The few sensory retraining studies that exist often examine different primary outcomes, with some reporting motor outcomes and some reporting somatosensory outcomes. There are two main issues with this. The first issue is that motor outcomes do not directly assess whether the somatosensory system has experienced recovery and/or has been rehabilitated. Here, we can make assumptions that some sensory recovery may occur, but it is necessary to have data to directly determine whether these improvements are occurring due to motor recovery, sensory recovery, or a combination of both. This issue speaks directly to our general lack of understanding of how sensory function recovers alongside motor function. The second issue is that for studies that do quantify somatosensory outcomes, the measurement tools used often poorly capture somatosensory function, are subjective, and are imprecise [83]. These factors make the interpretation of these studies and their relevance to rehabilitation difficult as there are no standard measures utilized in rehabilitation or clinical trials for assessing sensory function. Additionally, a recent review focusing on replicability of somatosensory interventions has suggested that many of the current studies lack sufficient detail for accurate replication for clinical implementation [84]. However, it is difficult to understand if these claims can be substantiated, since this review did not identify the randomized control trials (*n* = 61) that were examined.

#### Clinically-Focused Sensory Retraining

Work by Leeanne Carey and colleagues has also been some of the first to implement training protocols to improve somatosensory function. Their Study of Effectiveness of Neurorehabilitation on Sensation (SENSe) approach incorporates multi-modal sensory training, principles of feedback, graded difficulty, and focused sensory attention [61]. With this training framework, their group identified downstream effects of somatosensory training on improving self-reported functional arm usage using the Motor Activity Log (MAL) [85]. This work has shown functional improvements in somatosensation with intervention after stroke, mostly notably touch and proprioception [61]. Recent work has used this framework as inspiration to understand the effectiveness of somatosensory training compared to motor training after stroke [86, 87]. Carlsson and colleagues found positive effects of sensory retraining on improving tactile thresholds and self-reported arm use, compared to a task-specific training control group, which focused on motor training during functional tasks [87]. However, De Bruyn and colleagues compared motor-based and sensory-based therapies, and found that motor-based therapy was better for improving motor outcomes [86]. Together, these results suggest that, in principle, sensory-based therapies demonstrate effectiveness for promoting functional improvements, but that the gains that occur as a result of targeted sensory-based training may be less robust than those directly targeting motor execution. However, we must note that the training tasks in each of these studies likely do not isolate or discount sensory contributions from the described “motor” training. With this consideration, it is important to note that ecologically, sensory and motor function are intimately linked and that any clinical training approaches would likely benefit from assessment and training in both domains. However, as studies examining sensory recovery and training after stroke have been limited, the development of targeted and isolated training of sensory function may offer a unique window into a better understanding of the mechanisms governing impairment and recovery of the somatosensory system after stroke.

#### Promoting Sensory Recovery with Technology and Robotics

As described above, sensory training in the clinical domain has focused primarily on multi-modal training approaches, while approaches using technology and robotics have generally focused on retraining limb proprioception with the goal of improving motor function. When considering the design and approach of training paradigms it is critical to note that human therapists may inadvertently provide additional sensory cues, such as tactile information, that may offer unintended training cues or sensory information that can be used in a compensatory way. With this, technology and robotics often have an advantage over traditional approaches, as they can deliver repeated, identical movements to directly target training of proprioception of the paretic limb [62].

To date, much of the work focusing on training of the proprioceptive system has been conducted in control participants [88, 89]. These studies have described improvements in proprioceptive detection thresholds, as well as improvements in motor function and learning. This work has suggested that improvements in motor function from proprioceptive training arise potentially from enhanced perception of errors, altered proprioceptive signaling from the muscles, and/or enhanced functional connectivity between somatosensory and motor areas of the brain [88–91]. These studies provide a critical foundation from which to base approaches for improving proprioception, as this evidence demonstrates that the somatosensory system is flexible and capable of improving with training. To date, few clinical studies have focused on training proprioceptive function, likely due to imprecise methods and complications related to motor confounds. Robotics provide a unique and useful approach, as these devices are capable of passively moving the limb, wrist, or finger. This is particularly useful in individuals with limited use of the limb, as the robotic device can support the body against gravity and passively move the body part without the requirement of active motor control or execution. Further, task designs using robotics can easily be done in the absence of vision, which allows for the isolation of proprioception for both assessment and training after stroke without relying on compensatory strategies that may require vision [92–95].

Current work using technology-based or robotic training approaches have typically used methods that augment sensory signaling in some capacity, typically through error augmentation or vibrotactile stimulation [28, 96–102]. Broadly, studies that have examined proprioceptive-based training have tested protocols that range from a single-day up to 3.5 weeks, observing generally positive improvements on various proprioceptive measures. Notably, the vast majority of these studies have measured improvements in sensory function and/or motor control directly after training, which limits our understanding of whether or not these techniques are suitable for inducing long-term retention weeks or months after administration of training paradigms. Ingemanson and colleagues (2019) examined the impact of sensory training with robotically-enhanced proprioceptive feedback of the fingers. They found that sensory status was a significant predictor of training-based functional gains, as captured by behavioral and neuroanatomical measures [28]. Improvements in proprioceptive detection thresholds have been found in studies using bi-planar robots after a single session of training [96], as well as 3 weeks of training [97]. Additionally, a case study used similar methodology and found marked improvements in a proprioception and motor performance [98]. Yeh and colleagues (2021) adopted a slightly different approach and applied vibrotactile stimulation during a sensory training paradigm to determine if proprioceptive detection thresholds of the wrist could improve with repeated training. They found that with a 2-day training bout that stroke survivors had improved detection thresholds during immediate post-testing as well as retention tests 3 days later [99]. Other studies have specifically removed visual feedback with the purpose of upweighting reliance on proprioception during training protocols. In a recent study from our group, we have implemented a self-guided proprioceptive robotic training paradigm where stroke survivors use the less-affected arm to recalibrate positioning of the more-affected arm. Here, in this proof-of-concept study, we have found that within a single training session that stroke survivors significantly improved some aspects of proprioceptive function as well as motor function [101]. Another study used virtual reality in combination with a goal-directed reaching task where vision was occluded to maximize proprioceptive feedback [102]. This one-week training protocol observed reduced motor errors without vision during training and improvements in clinical assessments of arm function [102]. Additional work implemented active-assisted tasks to explicitly engage proprioception with and without the use of vision. While the authors note positive outcomes in motor control after training, this study did not capture proprioceptive outcomes making it unclear whether the training improved proprioception after stroke [103].

Overall, approaches using augmented proprioception, vibrotactile stimulation, and self-guided proprioceptive training have shown promising results for the improvement of not only proprioceptive function but motor function immediately after training; however, future work needs to be completed in larger sample sizes, for longer training periods, assessing various post-test timing intervals, and in participants that appropriately represent the heterogeneity of sensorimotor impairment after stroke to best understand the effectivity and retention of somatosensory training.

#### Somatosensory Stimulation Techniques for Promoting Somatosensory Recovery

For the past two decades, research in stroke rehabilitation has focused on the use of non-invasive electrical stimulation as a sensory-based tool to enhance motor recovery [104, 105]. Typically, this method directly applies cutaneous electrical stimulation to the “to-be” rehabilitated body part (upper limb, lower limb). While the definitive mechanism of action is unknown, this technique is thought to act via a strengthening of connections between primary somatosensory and primary motor cortices [106]. This subsequent increase in afferent signaling is assumed to alter efferent signals responsible for motor execution. While this method has been shown to be effective in improving short-term motor outcomes of the hand [104, 105], the effects of electrical stimulation on sensory recovery are less clear. Other work has been relatively inconclusive, as studies have found variability in functional improvements [107], improvements is somatosensory function with and without stimulation protocols [105, 108], and short-term but not sustained long-term improvements in limb sensation [108–110]. Recently, there has been promising effects on sensory recovery with non-invasive brain stimulation with repetitive transcranial magnetic stimulation (rTMS) of the primary somatosensory cortex (S1) [111]. Primary outcomes for sensory function were derived from the Nottingham Sensory Assessment, a clinical measure of somatosensory function. While the evaluation of sensory outcomes is critically important, it is unclear how meaningful the magnitude of change in these scores is because most clinical measures of somatosensory function do not have established minimally clinically important differences (MCIDs).

More recent work seeking to improve somatosensory function after stroke has focused on more invasive electrical stimulation approaches like vagus nerve stimulation (VNS) [112–115]. These advanced stimulation techniques have received widespread attention as they have demonstrated promising gains in motor function after stroke [112–114, 116, 117]. Despite these gains, it is unclear whether or not somatosensory improvements accompany functional motor improvements using VNS. A recent case study aimed to pair VNS with tactile training after stroke in an individual that had significant sensory loss. Within this individual, they found that VNS paired with tactile therapy significantly improved tactile sensation, proprioception, and stereognosis [115]. These improvements were sustained after the paired therapy sessions were completed, which is promising evidence for the role of VNS for promoting improvements in sensory function. A second study examined individuals with sensory impairments to see if VNS paired with repetitive, functional arm movements would improve touch and proprioception [118]. Here, improvements in sensory function were measured via the Upper Extremity Fugl-Meyer and moderate but not clinically significant improvements were observed. The interpretability of the magnitude or type of sensory improvements in this study is likely limited, as the sensory portion of the Fugl-Meyer is not appropriate for capturing detailed measurements related to sensory function after stroke [119]. In order to fully understand the scope of impairment and subsequent improvement with advanced stimulation techniques, study designs should consider using detailed somatosensory clinical measures or advanced assessment techniques like robotics [62]. Given the promising results for motor rehabilitation paired with VNS, it is clear that future work is needed to fully understand the potential benefit of techniques like VNS for promoting sensory recovery. However, we must note that not all motor rehabilitation techniques have translated positively to sensory intervention. For example, there have been efforts to study the effects of epidural stimulation of the cervical spinal cord for post-stroke paresis of the upper limb; however, limited data from this case study (*n* = 2) did not demonstrate sensory changes during or after the course of the intervention [117].

### Considerations for Effectively Promoting Recovery of Sensory Systems

Despite recent advances in assessment approaches for measuring sensory deficits and training approaches for targeted improvement of sensory deficits, there is still a considerable gap in our understanding of (1) the mechanisms of sensory recovery, (2) how to effectively treat sensory impairments and promote sensory recovery, and (3) whether the recovery of sensory function is critical to functional outcomes after stroke. Somatosensory deficits have widespread consequences on function after stroke, including negative associations with motor function [19], paretic arm use [19, 85, 87], ADL function [19], activity participation [120], length of hospital stay [121], and poorer rehabilitation outcomes [28]. To date, research has shown that enhancing proprioception during rehabilitative training can lead to improved motor gains, is an important predictor of motor outcomes [28, 122], and is necessary for full recovery of motor function [79]. These positive results speak to the importance of measuring and understanding sensory impairment after stroke, but understanding the mechanisms and relationship related to overall motor function during recovery and rehabilitation is still significantly limited.

When considering sensory recovery, a contrasting point of view may pose the following question: Is it actually important to evaluate sensation if motor function improves anyways? While improvement of motor function is often the ultimate goal of rehabilitation after stroke, it is critical to understand what insight can be gained from understanding sensory outcomes in addition to motor outcomes. Our current understanding suggests that sensory function can recover after stroke [14, 123–125], and that sensory recovery can vary depending on factors such as stroke severity [124]. Additionally, when we consider stroke recovery, there may be a considerable amount of overlap between the sensory and motor systems. However, in several studies, it has been observed that up to 42% of stroke survivors have mismatched sensory and motor recovery [77, 122, 123]. These ideas are further complicated due to the relative novelty of sensory assessments and training paradigms in neurorehabilitation, as sample sizes tend to be relatively small in order to establish “proof of concept” approaches prior to engaging in larger scale studies or even clinical trials. Further, the lack of standardization of sensory-based outcomes makes it difficult to interpret and generalize results across these few, but important studies. While much of the current evidence has focused on *if* improvements in sensory function are possible, there is still a significant gap in understanding whether long-term retention of sensory gains is feasible and whether these gains translate or generalize to different aspects of sensorimotor function. Additionally, there is a significant gap in our understand of exactly *how* somatosensory recovery directly benefits function after stroke. To address this, it is critical for the field to not only understand if and when sensory recovery can occur or benefit from treatment, but also to understand the neural mechanisms that are responsible for these changes after stroke.

Current and future studies focusing on sensory function after stroke, or other neural injury, will likely be able to provide important insight into the mechanisms that guide sensory recovery. However, we must note, that while several studies have focused on the application of sensory training to improve function, there is generally a lack of hypothesized mechanisms responsible for this process. While it is assumed that the sensory system likely follows the same purported spontaneous recovery mechanisms as motor function [78], specific studies examining sensory recovery are needed to provide evidence to support this assumption. Further, rehabilitation-driven functional improvements often continue to take place beyond this window, which requires examination of these systems on a functional level [126]. This is especially important considering that current evidence has clearly demonstrated a disconnect between recovery of sensory and motor function in some individuals after stroke [77, 95, 122, 123]. A limitation of some current sensory training studies is that these studies have typically assessed motor outcomes instead of motor and sensory outcomes [103, 107], making it unclear whether these techniques suitably improve sensory function, motor function, or a combination of the two systems. Importantly, some recent studies have demonstrated improvements in both motor and proprioceptive function as a result of proprioceptive training [28, 96–99, 101–103]. Arguably, the majority of motor execution-based training paradigms and therapies in stroke rehabilitation also inherently engage training or rehabilitation of the sensory system. Experimentally and therapeutically, these two systems are difficult to decouple, as they serve opposing, but complementary roles for the perception and action of movement. However, this fact further highlights the importance of understanding which system or combination of systems may be driving functional improvements throughout recovery and subsequent rehabilitation.

## Conclusions

Overall, it is important to consider evaluating sensory information and what additional information can be gained to design and guide rehabilitation. Through a more complete understanding of the mechanisms underlying improvement of sensory and motor outcomes, it is likely that we can more precisely guide rehabilitation interventions. Thoughtful experimental designs that allow for the decoupling and re-integration of the sensory and motor systems will help us achieve this goal. Additional studies focusing on the neural correlates of recovery in both systems are critical to identifying key avenues for potential interventions.

## Key References

Papers of particular interest, published recently, have been highlighted as:

- Ingemanson ML, Rowe JR, Chan V, Wolbrecht ET, Reinkensmeyer DJ, Cramer SC. Somatosensory system integrity explains differences in treatment response after stroke. Neurology. 2019;92:e1098–108.
-
  - This study reveals that proprioception can be an important predictor of functional rehabilitation gains, as captured by behavioral and neuroanatomical measures.
- Semrau JA, Herter TM, Scott SH, Dukelow SP. Examining Differences in Patterns of Sensory and Motor Recovery After Stroke With Robotics. Stroke. 2015;46:3459–69.
-
  - This study describes the independent recovery patterns of the sensory and motor systems for 30% of individuals after stroke.
- Zandvliet SB, Kwakkel G, Nijland RHM, van Wegen EEH, Meskers CGM. Is Recovery of Somatosensory Impairment Conditional for Upper-Limb Motor Recovery Early After Stroke? Neurorehabil Neural Repair. 2020;34:403–16.
-
  - This study describes somatosensory recovery as an important component for “full” motor recovery after stroke.
- Zbytniewska-Mégret M, Salzmann C, Kanzler CM, Hassa T, Gassert R, Lambercy O, et al. The Evolution of Hand Proprioceptive and Motor Impairments in the Sub-Acute Phase After Stroke. Neurorehabil Neural Repair. 2023;37:823–36.
-
  - This study reports that 42% of their sample had dissociated proprioceptive and motor recovery patterns after stroke. Yet, proprioceptive function was a factor in improved (motor) function at discharge.
- Ballardini G, Carlini G, Giannoni P, Scheidt RA, Nisky I, Casadio M. Tactile-STAR: A Novel Tactile STimulator And Recorder System for Evaluating and Improving Tactile Perception. Front Neurorobotics. 2018;12:12.
-
  - This study designs and tests novel technology that can be used for understanding tactile impairments after stroke.
- Turville M, Carey LM, Matyas TA, Blennerhassett J. Change in Functional Arm Use Is Associated With Somatosensory Skills After Sensory Retraining Poststroke. Am J Occup Ther. 2017;71:7103190070p1–9.
-
  - This study discusses the effects somatosensory training can have on self-reported paretic arm use.
- Vahdat S, Darainy M, Thiel A, Ostry DJ. A Single Session of Robot-Controlled Proprioceptive Training Modulates Functional Connectivity of Sensory Motor Networks and Improves Reaching Accuracy in Chronic Stroke. Neurorehabil Neural Repair. 2019;33:70–81.
-
  - This study reports improvements in proprioception, motor control, and functional connectivity within sensory motor networks in a single proprioceptive training session.
- Yeh I-L, Holst-Wolf J, Elangovan N, Cuppone AV, Lakshminarayan K, Capello L, et al. Effects of a robot-aided somatosensory training on proprioception and motor function in stroke survivors. J NeuroEngineering Rehabil. 2021;18:77.
-
  - This study demonstrates individuals after stroke can reduce proprioceptive thresholds with a two-day proprioceptive training paradigm.
- De Freitas Zanona A, Romeiro Da Silva AC, Do Rego Maciel AB, Gomes Do Nascimento LS, Bezerra Da Silva A, Bolognini N, et al. Somatosensory Cortex Repetitive Transcranial Magnetic Stimulation and Associative Sensory Stimulation of Peripheral Nerves Could Assist Motor and Sensory Recovery After Stroke. Front Hum Neurosci. 2022;16:860965.
-
  - This study indicates promising effects on sensory recovery with non-invasive brain stimulation with repetitive transcranial magnetic stimulation (rTMS) of the primary somatosensory cortex (S1).

## Data Availability

No datasets were generated or analysed during the current study.
